# Validating Brain Tumor Reporting and Data System (BT-RADS) as a Diagnostic Tool for Glioma Follow-Up after Surgery

**DOI:** 10.3390/biomedicines12040887

**Published:** 2024-04-17

**Authors:** Yassir Edrees Almalki, Mohammad Abd Alkhalik Basha, Maha Ibrahim Metwally, Nesma Adel Zeed, Mohamad Gamal Nada, Sharifa Khalid Alduraibi, Ahmed A. Morsy, Rawda Balata, Ahmed Z. Al Attar, Mona M. Amer, Mohamed Abd El-Aziz Mohamed Farag, Sameh Abdelaziz Aly, Ahmed M. Abdelkhalik Basha, Enas Mahmoud Hamed

**Affiliations:** 1 Division of Radiology, Department of Internal Medicine, Medical College, Najran University, Najran 61441, Saudi Arabia; yealmalki@nu.edu.sa; 2 Department of Diagnostic Radiology, Faculty of Human Medicine, Zagazig University, Zagazig 44519, Egypt; mimetwally@zu.edu.eg (M.I.M.); nonyzeed1984@gmail.com (N.A.Z.); mohammedgamalnada.mn@gmail.com (M.G.N.); enashamedmya@gmail.com (E.M.H.); 3 Department of Radiology, College of Medicine, Qassim University, Buraidah 52571, Saudi Arabia; salduraibi@qu.edu.sa; 4 Department of Neurosurgery, Faculty of Human Medicine, Zagazig University, Zagazig 44519, Egypt; dr.ahmed.ali.morsy@gmail.com; 5 Department of Clinical Oncology and Nuclear Medicine, Faculty of Human Medicine, Zagazig University, Zagazig 44519, Egypt; drrawdabalata@gmail.com (R.B.); ahmedalata982@gmail.com (A.Z.A.A.); 6 Department of Neurology, Faculty of Human Medicine, Zagazig University, Zagazig 44519, Egypt; monaamer2345@gmail.com; 7 Department of Radio-Diagnosis, Faculty of Human Medicine, Al Azhar University, Cairo 11884, Egypt; mabdelaziz43@yahoo.com; 8 Department of Diagnostic Radiology, Faculty of Human Medicine, Benha University, Benha 13511, Egypt; drsamehaly75@gmail.com; 9 Faculty of General Medicine, Saint Petersburg State University, Egypt Branch, Cairo 11646, Egypt; ahm7edbasha@gmail.com

**Keywords:** glioma, magnetic resonance imaging, BT-RADS, follow-up, tumor progression

## Abstract

Gliomas are a type of brain tumor that requires accurate monitoring for progression following surgery. The Brain Tumor Reporting and Data System (BT-RADS) has emerged as a potential tool for improving diagnostic accuracy and reducing the need for repeated operations. This prospective multicenter study aimed to evaluate the diagnostic accuracy and reliability of BT-RADS in predicting tumor progression (TP) in postoperative glioma patients and evaluate its acceptance in clinical practice. The study enrolled patients with a history of partial or complete resection of high-grade glioma. All patients underwent two consecutive follow-up brain MRI examinations. Five neuroradiologists independently evaluated the MRI examinations using the BT-RADS. The diagnostic accuracy of the BT-RADS for predicting TP was calculated using histopathology after reoperation and clinical and imaging follow-up as reference standards. Reliability based on inter-reader agreement (IRA) was assessed using kappa statistics. Reader acceptance was evaluated using a short survey. The final analysis included 73 patients (male, 67.1%; female, 32.9%; mean age, 43.2 ± 12.9 years; age range, 31–67 years); 47.9% showed TP, and 52.1% showed no TP. According to readers, TP was observed in 25–41.7% of BT-3a, 61.5–88.9% of BT-3b, 75–90.9% of BT-3c, and 91.7–100% of BT-RADS-4. Considering >BT-RADS-3a as a cutoff value for TP, the sensitivity, specificity, and accuracy of the BT-RADS were 68.6–85.7%, 84.2–92.1%, and 78.1–86.3%, respectively, according to the reader. The overall IRA was good (κ = 0.75) for the final BT-RADS classification and very good for detecting new lesions (κ = 0.89). The readers completely agreed with the statement “the application of the BT-RADS should be encouraged” (score = 25). The BT-RADS has good diagnostic accuracy and reliability for predicting TP in postoperative glioma patients. However, BT-RADS 3 needs further improvements to increase its diagnostic accuracy.

## 1. Introduction

Glioma is the most common primary brain tumor, with the highest grade, grade IV glioma, or glioblastoma, representing the most common form in adults and having a particularly poor prognosis [1]. There are multimodal treatments, including radiotherapy, chemotherapy, and surgery; however, the results remain inadequate [2,3]. The poor prognosis associated with glioblastoma emphasizes the need for precise monitoring of tumor progression (TP) and treatment response [4,5].

Magnetic resonance imaging (MRI) is essential for assessing brain tumors during preoperative and postoperative periods [5,6]. Brain tumor MRI findings, however, are notably challenging to interpret since true TP may look similar to treatment effects, leading to ambiguous interpretations and difficult management decisions. Consequently, it is challenging for radiologists to assess the treatment response of brain tumors [5,7,8]. There are currently several guidelines for assessing the therapeutic response of brain tumors, including the World Health Organization, Levin, MacDonald, and Response Assessment in Neuro-Oncology (RANO), which are more often used in clinical trials. Due to reasons such as complexity, requirement for repeated measures, significant interobserver variability, and physician misunderstanding, their use in radiology has been limited to date [5,7,9,10,11,12]. To address these shortcomings in current practice, a team of neuroradiologists, neuro-oncologists, neurosurgeons, and radiation oncologists created the Brain Tumor Reporting and Data System (BT-RADS) in 2018 [13]. 

A BT-RADS is a structured glioma surveillance reporting system that uses MR imaging patterns, clinical evaluation, and therapeutic scheduling to give each report a numeric category from 0 to 4 based on the possibility of TP. Each category is associated with a specific management recommendation. Categorization is based on the changes in four MR imaging patterns: enhancing components, FLAIR components, mass effects, and new lesions compared with the most recent prior brain MRI [12,13,14]. In this scenario, the BT-RADS has been shown to improve the clarity, consistency, and confidence ratings of referral providers in radiology reports and facilitate patient management decisions [15]. Although qualitative and quantitative improvements for the BT-RADS have been reported [14,15,16], the complete value of the BT-RADS still needs to be evaluated. Subsequently, we carried out this multi-institutional prospective study to assess the diagnostic accuracy and reliability of the BT-RADS in predicting TP in patients with post-treated glioma. Furthermore, we conducted a simple survey to assess readers’ agreement with this classification.

## 2. Methods

### 2.1. Ethical Statement

The study was authorized by the Institutional Review Board (approval number: ZU-9857), and all participants signed informed consent. The study was conducted in accordance with the principles of the Declaration of Helsinki. 

### 2.2. Eligibility Criteria 

Between October 2021 and August 2023, we identified 90 postoperative glioma patients at three academic institutions. The inclusion criteria were (i) age ≥ 18 years and (ii) recent total or subtotal high-grade glioma (HGG) resection. Once enrolled, all patients underwent a series of follow-up MRI scans. The exclusion criteria were (i) patients categorized as BT-RADS-0 (n = 5), (ii) patients lost during the follow-up period (n = 7), (iii) patients with poor-quality contrast-enhanced MR images (n = 2), and (iv) patients with postoperative surgical bed ischemic insult (n = 3). 

### 2.3. Postoperative Clinical Assessment

All patients were clinically evaluated by neurosurgeons with >10 years of clinical experience. All neurosurgeons were blinded to the imaging findings but assessed the clinical status of patients according to the Neurological Assessment in Neuro-Oncology (NANO) scale [17]. Nine neurological domains were tested for each patient: upper extremity ataxia, visual field, strength, facial strength, gait, language, sensation, behavior, and degree of consciousness. A score range of 0–2 was given to each domain. Finally, neurosurgeons reported that each patient’s clinical state was clinically stable, improved, or worsened.

### 2.4. MRI Examination

Brain MRI examinations were conducted using a 1.5-T MRI system (Optima 450 GEM, GE Healthcare, Chicago, IL, USA, and Achieva class IIa, Philips Medical Systems, Best, Netherlands) with an 8-channel head coil. The acquired MRI sequences included axial fluid attenuation inversion recovery (FLAIR) (TE = 130, TR = 8500, TI = 2509, FOV = 24, matrix = 256 × 192, slice thickness = 5, bandwidth = 31.25), axial fast release fast spin echo (FrFSE) T2WI (TE = 102, TR = 4430, FOV = 24, matrix = 256 × 224, slice thickness = 5, bandwidth = 31.25), coronal FrFSE T2WI (TE = 120, TR = 4597, FOV = 22, matrix = 320 × 224, slice thickness = 2, bandwidth = 22.73), and axial pre-contrast and multiplanar post-contrast FSE T1WI (TE = Min Full, TR = 480, FOV = 24, matrix = 192 × 288, slice thickness = 5) after intravenous injection of 0.1 mmol/kg of gadopentetate dimeglumine (2 mL/s).

### 2.5. MRI Image Analysis

Images were transferred to workstations and analyzed using a dedicated platform-extended workstation (Advantage Workstation, GE Healthcare, Chicago, Illinois, United States, Philips Medical System, Best, The Netherlands, or PaxeraUltima, Paxera Viewer version 5.0.9.6, PaxeraHealth, Newtone, MA, USA). Five neuroradiologists (Y.E.A., M.I.M., N.A.Z., E.M.H., and M.G.N. with 16, 15, 14, 14, and 12 years of experience in neuroimaging, respectively) independently evaluated the initial two consecutive MR images. Radiologists were aware of the patients’ preoperative images, operative data, received treatment, and clinical states. The radiologists received three hours of detailed education on the BT-RADS before the start of the study, which included 20 practical cases chosen from a different time period than the study population. On the MRI images, the following features were assessed for each postoperative glioma: enhancing component (unchanged, decrease, increase <25%, or increase >25%), FLAIR component (unchanged, decrease, increase <25%, or increase >25%), mass effect (unchanged, decrease, or increase), and new lesion (no, yes (indeterminant), or yes (definite)). Finally, each radiologist independently assigned a BT-RADS category for each postoperative glioma using the BT-RADS developed by Weinberg et al. [13] to predict TP based on MRI features and the clinical state of the patients. The BT-RADS categories are described in Table 1.

### 2.6. Reference Standards

The final diagnosis of TP was established based on several factors. The first factor was (a) clinical and imaging follow-up (n = 46): each patient had serial follow-up MRIs (at least two) after the initial two consecutive MRIs. TP was based on follow-up MRIs reviewed by a multidisciplinary brain tumor team, including neuroradiology, neuro-oncology, radiation oncology, and neurosurgery members. The team was blinded to the patients’ previous imaging findings and clinical data. The neuroradiology team consisted of two neuroradiologists (S.A.A. and M.A.A.B. with over 20 years of experience in neuroimaging). They consensually assessed the follow-up MRI findings. Clinical follow-up was performed according to the NANO scale. TP was defined as a progressive increase in enhancing or FLAIR component, increased mass effect, or the appearance of a new lesion on follow-up MRIs. Non-progression was defined as the stability or improvement of lesions on follow-up MRIs for >6 months [18,19]. (b) The second factor was histopathological examination after repeating surgical resection or stereotactic biopsy (n = 27): biopsy-proven TP was required if imaging findings showed definite TP. An experienced neuropathologist blinded to the imaging findings reported the pathological results. More than 75% of tumor cells in tissue specimens indicated TP, whereas none or scanty tumor cells (<25%) indicated non-progression [20].

### 2.7. Reader Acceptance

The radiologists were asked to complete a brief survey after reviewing all MR images to assess BT-RADS acceptance. A 5-point ordinal scale was used to evaluate the response (one = strongly disagree; two = disagree; three = neither agree nor disagree; four = agree; five = strongly agree). The scores were calculated as the sum of each reader’s points.

### 2.8. Statistical Analysis

The collected data were analyzed using SPSS version 26 (IBM, Armonk, NY, USA) or MedCalc version 15.8 (Mariakerke, Belgium). Continuous variables were described using means and standard deviations, whereas categorical variables were described using numbers and percentages. Using the receiver operating characteristic (ROC) curve, we established the best cutoff value of the BT-RADS that predicted TP and calculated its diagnostic accuracy based on imaging, clinical follow-up, and pathological results. The agreement between the radiologists regarding the final BT-RADS category and MR features was calculated using kappa (κ) statistics. A κ value of 0.00–0.20 indicated poor agreement; 0.21–0.40 indicated fair agreement; 0.41–0.60 indicated moderate agreement; 0.61–0.80 indicated good agreement; 0.81–1.00 indicated very good agreement. Statistical significance was determined by a *p*-value of 0.05.

## 3. Results

### 3.1. Patients and Glioma Characteristics

**Figure 1:**
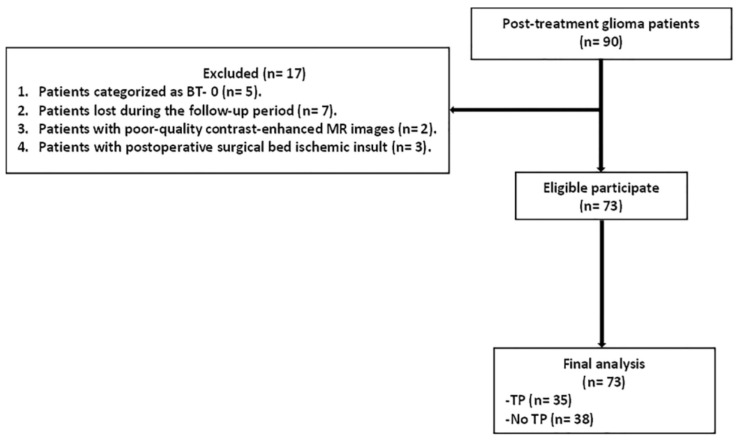
Flowchart of the study.

The final analysis of our study involved 73 patients (male, 67.1%; female, 32.9%; mean age, 43.2 ± 12.9; age range, 31–67 years) with 322 MRI examinations. The clinicopathological features of the patients and their gliomas are summarized in Table 2. The mean follow-up period of the study was 15.3 ± 4.8 months (range, 8–25 months). The mean interval to the first postoperative MRI scan was 4.3 ± 1.5 months (range, 1–10 months). The mean interval between the first and second postoperative MRI scans was 3.8± 0.9 months (2–7 months). Grade III gliomas were pathologically confirmed in 31 patients, whereas 42 patients had grade IV gliomas. Twenty-seven patients underwent histopathological examination after stereotactic biopsy or repeat surgery, and 42 underwent clinical and imaging follow-up. The final assessment of gliomas showed 35 patients with TP and 38 without TP. Figure 1 demonstrates the flowchart of the study.

### 3.2. Distributions of BT-RADS Categories

Table 3 shows the frequency distributions of the assigned BT-RADS categories after the initial two postoperative MRIs, stratified by reader and final diagnosis (TP or no TP). Across the five readers, the number of gliomas assigned to each category ranged as follows: 7–11 for BT-RADS-1a, 9–12 for BT-RADS-1b, 12–14 for BT-RADS-2, 4–12 for BT-RADS-3a, 8–15 for BT-RADS-3b, 4–11 for BT-RADS-3c, and 7–17 for BT-RADS-4.

### 3.3. Incidence of TP across BT-RADS Categories

The incidence of pathologically or clinically confirmed TP increased with higher assigned BT-RADS categories (Table 3). According to the readers, the proportion of patients with TP ranged from 0–18.2% for BT-RADS-1a, 10–25% for BT-RADS-1b, 14.3–30.8% for BT-RADS-2, 25–41.7% for BT-RADS-3a, 61.5–88.9% for BT-RADS-3b, 75–90.9% for BT-RADS-3c, and 91.7–100% for BT-RADS-4.

### 3.4. Diagnostic Accuracy of BT-RADS

Table 4 presents the ROC analysis using different BT-RADS cutoff categories to predict TP status. ROC analysis demonstrated that the BT-RASDS classification had lower sensitivities with the worsening categories (BT-RADS-3 and 4), and the best cutoff value for predicting TP was the BT-RADS-3a category or higher. According to the reader, when >BT-RADS-3a was used as a predictor for TP, the sensitivity, specificity, accuracy, positive predictive value (PPV), and negative predictive value (NPV) of the BT-RADS were 68.6–85.7%, 84.2–92.1%, 78.1–86.3%, 80.6–89.7%, and 75.6–86.8%, respectively (Table 5).

### 3.5. Inter-Reader Reliability of BT-RADS

The inter-reader agreement (IRA) for evaluating the key MRI features and assigning the final BT-RADS category is summarized in Table 6. Overall, the IRA was very good for identifying new lesions (κ = 0.89) and good for evaluating the FLAIR component (κ = 0.67), mass effect (κ = 0.69), and determining the final BT-RADS classification (κ = 0.75). The agreement was moderate for assessing the enhancing component (κ = 0.54).

### 3.6. Reader Acceptance of BT-RADS

Table 7 shows the readers’ responses to the survey statements about implementing the BT-RADS in clinical practice. The readers strongly agreed that “the application of BT-RADS should be encouraged” (score = 25) and that “reporting of post-treatment glioma imaging should follow a structured format” (score = 24). They also indicated that “BT-RADS needs further modification” (score = 24). There was a moderately high agreement with statements like “the structured reporting template of BT-RADS maintains consistency of reports” (score = 24) and “the application of BT-RADS constrains radiologists’ and clinicians’ communication” (score = 23). However, the readers gave lower scores to statements suggesting BT-RADS currently provides clarity, with “radiology reports following BT-RADS clarify significant findings” (score = 17. The lowest agreement was with the statement “BT-RADS is equivalent to other ACR reporting systems, as BI-RADS and LI-RADS” (score = 14).

**Figure 2:**
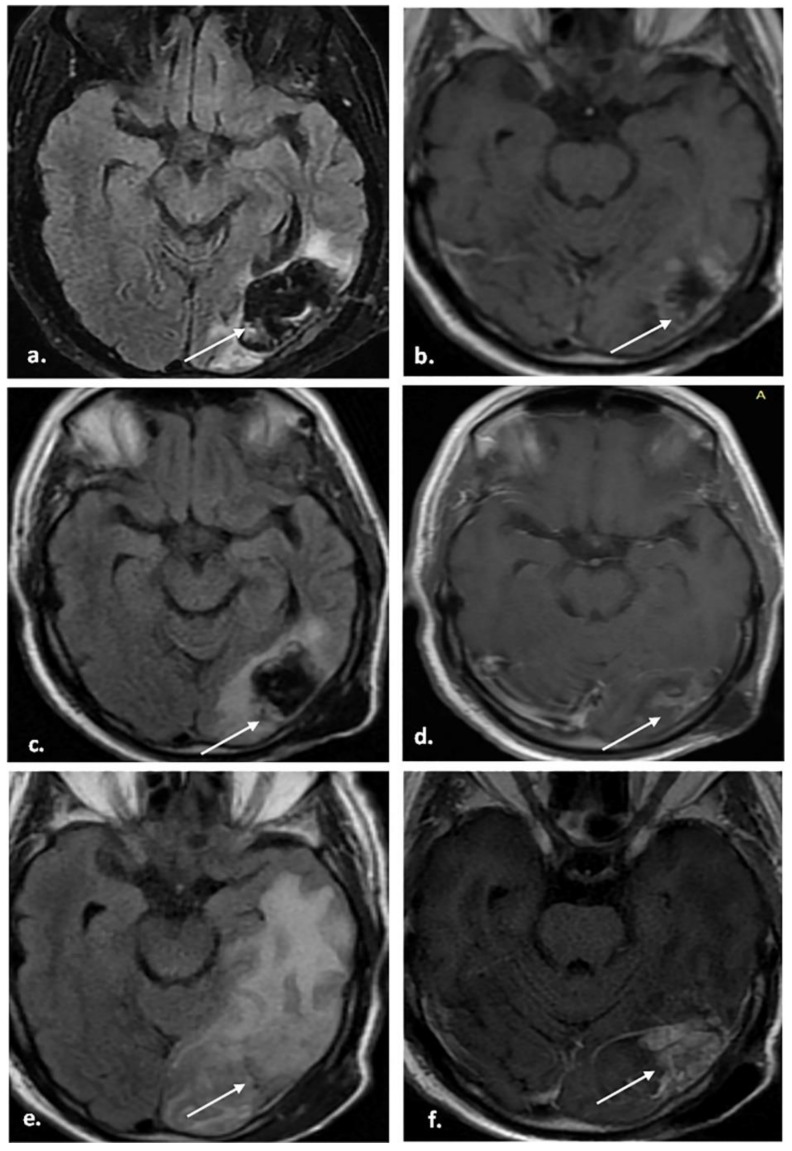
A 67-year-old male patient underwent total resection of pathologically proven HGG. The patient did not receive any antiangiogenic treatment. (**a**,**b**) MRI was performed 2 months after the operation while the patient was undergoing radiotherapy. (**a**) Axial FLAIR shows a CSF-like signal intensity resection cavity (arrow). (**b**) The axial postcontrast T1WI shows a mild peripheral enhancement of the cavity (arrow). (**c**,**d**) A follow-up MRI was performed 45 days after the completion of radiotherapy; the patient was clinically stable. (**c**) The axial FLAIR image shows an increased signal at the periphery of the resection cavity (arrow). (**d**) The axial post-contrast T1WI shows patchy central enhancement with less peripheral enhancement (arrow). The patient was categorized as BT-RADS-3a. (**e**,**f**) A follow-up MRI was performed 6.5 months after the completion of radiotherapy; the patient was clinically deteriorating. (**e**) The axial FLAIR shows a new intermediate signal-intensity lesion (arrow) with surrounding edema and mass effect. (**f**) The axial post-contrast T1WI image shows heterogeneous enhancement of the lesion (arrow). The multidisciplinary brain tumor team considered tumor progression, the patient underwent reoperation, and histopathology showed recurrence.

**Figure 3:**
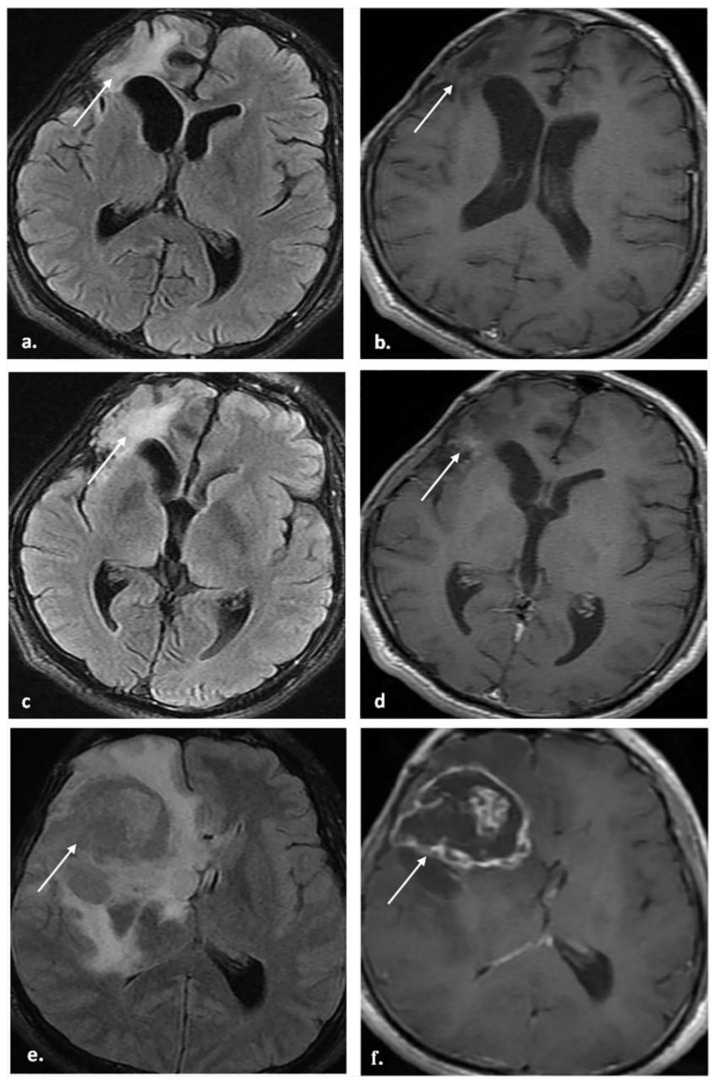
A 31-year-old male patient underwent total resection of HGG. The patient did not receive antiangiogenic therapy. (**a**,**b**) MRI was performed 9 months after completion of radiotherapy. (**a**) Axial FLAIR image shows right frontal high-signal intensity gliosis at the operative bed (arrow) with a negative mass effect on the ipsilateral ventricular system. (**b**) The axial post-contrast T1WI image shows no enhancement (arrow). (**c**,**d**) A follow-up MRI was performed 15 months after the completion of radiotherapy; the patient was clinically stable. (**c**) The axial FLAIR image shows stable high-signal intensity gliosis (arrow) with decreased negative mass effect on the ventricular system. (**d**) The axial post-contrast T1WI image shows small, enhanced foci (arrow). The patient was categorized as BT-RADS-3b. (**e**,**f**) A follow-up MRI was performed 22 months after the completion of radiotherapy; the patient was clinically deteriorating. (**e**) The axial FLAIR image shows a new intermediate signal intensity lesion (arrow) with a positive mass effect on the ipsilateral ventricular system. (**f**) The axial post-contrast T1WI image shows heterogeneous marginal enhancement of the lesion (arrow). The multidisciplinary brain tumor team considered tumor progression, the patient underwent reoperation, and histopathology showed recurrence.

**Figure 4:**
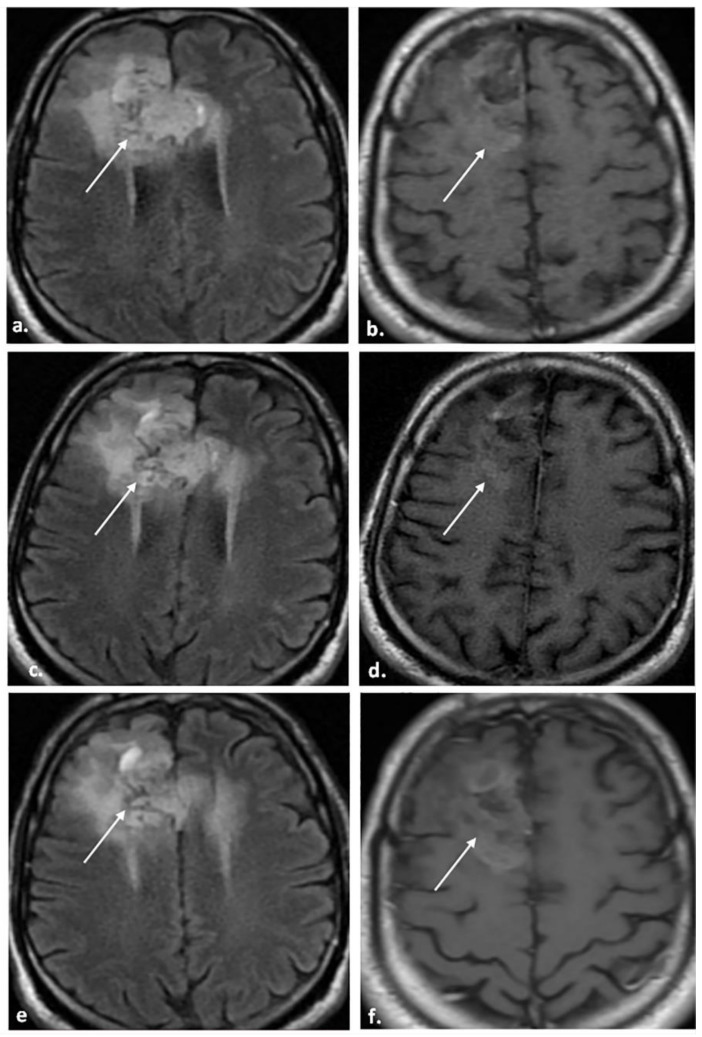
A 50-year-old male patient underwent total resection of HGG. The patient did not receive antiangiogenic therapy. (**a**,**b**) MRI was performed 4 months after the completion of radiotherapy. (**a**) The axial FLAIR image shows a right frontal high signal intensity area at the operative bed (arrow) extending to the genu of the corpus callosum to the opposite side with a mild positive mass effect on the related cortical sulci. (**b**) The axial post-contrast T1WI image shows no enhancement (arrow). (**c**,**d**) A follow-up MRI was performed 8 months after the completion of radiotherapy; the patient was clinically stable. (**c**) The axial FLAIR image shows a stable FLAIR component and mass effect (arrow). (**d**) The axial post-contrast T1WI image shows a stable enhancement pattern (arrow). The patient was categorized as BT-RADS-2. (**e**,**f**) A follow-up MRI was performed 12 months after the completion of radiotherapy; the patient was clinically stable. (**e**) The axial FLAIR image shows a stable FLAIR component and no mass effect (arrow). (**f**) The axial post-contrast T1WI image shows a stable enhancement pattern (arrow). The multidisciplinary brain tumor team considered the lesion to be non-progressive.

Figure 2, Figure 3 and Figure 4 represent some cases from our study.

## 4. Discussion

Understanding the BT-RADS lexicon and how to apply it accurately can improve care in daily practice by reducing bias in imaging assessment, promoting the prediction of glioma progression, and avoiding unnecessary repeated operations or alterations in treatment. The present prospective multicenter study provides important novel insights into the diagnostic accuracy, reliability, and clinical acceptance of using the BT-RADS structured reporting system for postoperative monitoring of glioma patients. To our knowledge, this is one of the largest evaluations of the BT-RADS to date, with 73 patients and over 300 MRI examinations interpreted by 5 independent experienced neuroradiologists. A key novel finding is that the BT-RADS demonstrates good diagnostic accuracy for predicting TP when using a cutoff value of >BT-RADS 3a. Across readers, the sensitivity ranged from 68.6 to 85.7% and specificity from 84.2 to 92.1%, with an overall accuracy of 78.1 to 86.3%. These performance characteristics support the utility of the BT-RADS as a clinical tool to reliably identify patients requiring changes in management due to TP versus those who can continue routine monitoring. A recent study published by Kim et al. [21] validated the prognostic role of the BT-RADS and reported that the BT-RADS with the highest probability of worsening at the subsequent follow-up was BT-RADS-3b. A retrospective study by Yang et al. [22] reported that the BT-RADS with conventional MRI yielded a sensitivity of 88%, specificity of 55%, and accuracy of 74% for differentiating TP from non-progression in postoperative high-grade glioma (HGG) patients with BT-RADS-3 lesions. Another recent prospective study assessed the accuracy of BT-RADS-3 in detecting TP and showed 76.9% sensitivity, 64.3% specificity, and 70.4% accuracy [23].

The current study showed that the BT-RADS classification system has limited sensitivity for predicting TP in postoperative gliomas, specifically for worsening categories (68.6–85.7% for BT-RADS-3a, 45.7–54.3% for BT-RADS-3b, and 17.1–45.7% for BT-RADS-3c). This finding is not surprising, as the BT-RADS depends on conventional MRI, which previous studies have shown to be ineffective in distinguishing TP from non-progression [24,25,26,27]. A meta-analysis by van Dijken et al. [24] concluded that conventional MRI is unreliable for assessing treatment response in HGG, with a sensitivity of 68% and a specificity of 77%.

As a result of the limited sensitivity of the BT-RADS in predicting TP, particularly with the worsening categories (BT-RADS-3), some authors [22,23] have tried to enhance the diagnostic power of the BT-RADS with the incorporation of advanced MR imaging techniques. Yang et al. [22] found that adding DWI and perfusion-weighted imaging (PWI) to the BT-RADS can considerably enhance the diagnostic accuracy for distinguishing TP from non-progression in postoperative HGG patients with a sensitivity of 98%, a specificity of 85%, and an accuracy of 95%. Also, Metwally et al. [23] concluded that the sensitivity of BT-RADS-3 for predicting TP increased after adding DWI to the BT-RADS. A meta-analysis without the BT-RADS conducted by van Dijken et al. [24] proved that advanced MRI techniques are more accurate than conventional MRI in differentiating TP from non-progression. However, because of the cost burden, different acquisition techniques, and non-routinely requested PWI in radiological centers, we recommend adding DWI to the BT-RADS and restricting other advanced MR techniques, such as PWI, to doubted cases when there is still uncertainty of the BT-RADS category after using conventional MRI and DWI.

We assessed the incidence of TP and found that it increased with increasing a BT-RADS category. According to our readers, TP was noted in 25–41.7% of patients classified as BT-RADS-3a, 61.5–88.9% of patients classified as BT-3b, 75–90.9% of patients classified as BT-3c, and 91.7–100% of patients classified as BT-RADS-4. According to Yang et al. [22], the recurrence rate for BT-RADS-3a was 21.4%, for BT-RADS-3b was 61.5%, and for BT-RADS-3c was 78.4%.

We comprehensively evaluated the inter-reader reliability of the BT-RADS application across multiple key imaging features. The results revealed very good agreement for identifying new lesions (κ = 0.89) and good agreement for assessing FLAIR changes (κ = 0.67), mass effect (κ = 0.69), and determining the final BT-RADS categorization (κ = 0.75). This demonstrates that the BT-RADS criteria can be applied consistently by different radiologists, which is essential for generalizability across practices. These results are similar to those of Parillo et al. [28], who concluded that the BT-RADS had good inter-rater reliability with a high agreement rate among expert radiologists and radiologists in training. Cooper et al. [29] reported that the overall agreement rate between primary and secondary reviews was 82.2%, with perfect agreement for studies with improvement categories (BT-RADS-1a or BT-RADS-1b) and lower levels of agreement for studies with worsening categories (BT-RADS-3a and BT-RADS-4). Additionally, we reported IRA for MR features of gliomas. The overall IRA was moderate for the enhancing component (κ = 0.54), good for the FLAIR component (κ = 0.67) and mass effect (κ = 0.69), and very good for new lesions (κ = 0.89). However, to date, no studies have assessed the IRA of such features in the BT-RADS. Thus, our results are not directly comparable to the literature.

Implementing a BT-RADS at a large university hospital enhanced radiology report perceptions among referring physicians and radiologists by improving report consistency and communication and assisting decision-making and research [15,16]. We assessed our readers’ acceptance of the routine use of the BT-RADS in reporting postoperative glioma imaging. A relatively lower score was given for “radiology reports following BT-RADS clarify significant findings”. Our readers explained this by deficient data on glioma extension regarding the cross to the opposite side, corpus callosum involvement, transependymal spread, and the lack of diffusion criteria and metabolite assessment by MRS. Additionally, “BT-RADS is equivalent to other ACR reporting systems, as BI-RADS and LI-RADS” was given a lower score as the BT-RADS, unlike other RAD systems, is a classification system whose objective is to monitor patient surveillance rather than to provide a diagnosis. Moreover, the value of the BT-RADS cannot be based on a single MRI examination but on multiple follow-up examinations. However, “The application of BT-RADS should be encouraged” and “post-treatment glioma imaging should follow a structured format” received high scores.

While multidisciplinary teams remain crucial for determining diagnosis and treatment plans for brain tumor patients, the BT-RADS can be a valuable complementary tool in the clinical setting. Its potential applications include integrating multidisciplinary tumor board discussions and utilizing its structured reporting and standardized lexicon to facilitate clear communication among specialists. Additionally, BT-RADS’ comprehensive assessment, incorporating advanced imaging techniques, can aid in evaluating treatment response, particularly in distinguishing true TP from treatment-related changes in high-grade glioma patients undergoing multimodal therapies. Adopting the BT-RADS can lead to more standardized and consistent imaging reports, benefiting large healthcare systems and patient transfers. Furthermore, incorporating the BT-RADS into training curricula for radiologists, neuro-oncologists, and other specialists can foster interdisciplinary collaboration and communication from an early stage. While not replacing multidisciplinary decision-making, the BT-RADS can enhance clinical workflows by improving communication, treatment response evaluation, and standardization across different healthcare settings.

RANO (Response Assessment in Neuro-Oncology) criteria were established in 2010 as guidelines for assessing treatment response in clinical trials for high-grade gliomas. RANO mainly focuses on measuring contrast-enhancing tumor portions on MRI [30]. While RANO is valuable for standardizing response evaluation in trials, it has limitations in clinical practice, requiring a more comprehensive assessment. The BT-RADS serves as a complementary tool by providing a structured reporting system with standardized terminology to describe overall brain tumor imaging findings beyond just contrast enhancement. The BT-RADS incorporates evaluations of tumor morphology, the extent of surrounding edema/mass effect, and patterns of enhancing and non-enhancing tumor components. It uses these factors to generate a scoring system that estimates the likelihood of tumor type and behavior [14]. Rather than replacing RANO’s focus on treatment response metrics, the BT-RADS aims to comprehensively characterize and communicate the full range of imaging findings to facilitate optimal multidisciplinary treatment planning alongside criteria like RANO. Its structured lexicon promotes clear communication among the cancer care team [16].

Our findings clearly encourage the application of the BT-RADS in postoperative glioma imaging. However, the BT-RADS still needs some improvements to increase its diagnostic accuracy and become useful and comprehensive for all pertinent descriptors and definitions. The key to these improvements may include the incorporation of DWI into the BT-RADS, the use of other advanced MR techniques in doubted cases when there is still uncertainty of the BT-RADS category, a more descriptive analysis of TP regarding its relation to the corpus callosum and ependymal surface of the ventricular system, and the assessment of the infiltrative nature of the glioma in the surrounding apparently normal brain parenchyma, mainly by using advanced imaging modalities such as MRS and PWI.

Finally, this study makes several key novel contributions to the literature on BT-RADS validation and clinical implementation for postoperative glioma monitoring: (1) establishing the diagnostic accuracy benchmarks across multiple readers, (2) demonstrating good inter-reader reliability, and (3) providing insights from radiologists’ perspective on clinical acceptance and potential areas for improvement. These findings support the increasing adoption of the BT-RADS while underscoring the need for continued refinements to maximize its clinical utility.

A notable strength of our study is the prospective design, which evaluates BT-RADS performance on actual clinical cases rather than retrospective assignments. Moreover, the multi-institutional nature increases the generalizability of the findings. Additionally, the study provides novel insights into radiologists’ perspectives on the BT-RADS through the reader acceptance survey.

## 5. Limitations

This study was subjected to several limitations: (1) Sample size: Though adequate for preliminary exploration of the BT-RADS in the postoperative glioma monitoring, it may limit our findings’ generalizability. (2) Prognostic evaluation: The role of the BT-RADS as a prognostic tool for overall survival in glioma patients was not assessed, marking a potential area for future research. (3) Expertise in image evaluation: The evaluation of all MR images was conducted by experienced radiologists, which might influence the perceived diagnostic accuracy of the BT-RADS. (4) Diagnosis confirmation: Not all diagnoses were confirmed via tissue pathology; more than half of the patients (63%) were confirmed by clinical and imaging follow-up, which could affect the validity of our findings. (5) Glioma subtypes: The study did not differentiate among various types of gliomas for sub-analyses due to the relatively small sample size. This limitation underscores the need for larger cohort studies to explore BT-RADS’ performance across different glioma subtypes. (6) Imaging technology: The study exclusively used a 1.5 Tesla MRI for all examinations, which may not reflect the diagnostic potential of higher-field MRIs. (7) Clinician familiarity with the BT-RADS: The adoption and effectiveness of the BT-RADS may be hindered by its relatively new introduction and current unfamiliarity among many clinicians. Addressing these limitations will be crucial for enhancing the applicability and accuracy of the BT-RADS in clinical settings and for future research aimed at refining glioma management strategies.

## 6. Conclusions

The current study indicates that the BT-RADS has good diagnostic accuracy and reliability for predicting TP in postoperative gliomas. However, the BT-RADS-3 needs further improvements to increase its diagnostic accuracy.

## Figures and Tables

**Table 1:** BT-RADS categories and description.

Category	Description	Imaging Patterns	Management Recommendation
BT-RADS-0	No score.	New baseline, incomplete study, or inability to categorize.	Continued follow-up, no change.
BT-RADS-1a	Improvement suspected due to a decrease in TP and/or treatment effects. Clinically stable or improved.	Reduction in enhancing component, FLAIR component, mass effect, or resolution of lesions compared with prior MRI.	Continued follow-up, no change.
BT-RADS-1b	Improvement potentially due to treatment effect. Clinically stable or improved.	Reduction in enhancing component, FLAIR component, mass effect, or resolution of lesions compared with prior MRI.	Continued follow-up, no change.
BT-RADS-2	No significant change. Clinically stable.	No substantial change in enhancing component, FLAIR component, mass effect, or new lesions compared with prior MRI.	Continued follow-up, no change.
BT-RADS-3a	Worsening may represent the treatment effect. Clinically stable.	Mild increase (<25%) in enhancing component, FLAIR component, or mass effect compared with prior MRI.	Decreased time interval of follow-up.
BT-RADS-3b	Indeterminate. Worsening may be a mix of TP and treatment effects. Clinically stable.	Moderate increase in enhancing component, FLAIR component, mass effect, or new lesion compared with prior MRI.	Decreased time interval of follow-up.
BT-RADS-3c	Worsening favors TP. Clinically worsening.	Significant increase (>25%) in enhancing component, FLAIR component, mass effect, or definite new lesion compared with prior MRI.	Change in management vs. decreased time interval of follow-up.
BT-RADS-4	Worsening, highly suspicious for TP. Clinically worsening.	Substantial increase in enhancing component, FLAIR component, mass effect, and/or multiple new lesions compared with prior MRI.	Change in management.

MRI = magnetic resonance imaging; TP = tumor progression; FLAIR = Fluid attenuated inversion recovery.

**Table 2:** Clinicopathologic characteristics of patients and gliomas.

Characteristic	Value
Total No. of patients	73
Age, (years), mean ± SD (range)	43.2 ± 12.9 (31–67)
Sex	
Male	49 (67.1)
Female	24 (32.9)
Total No. of MRI	322
Follow-up period (months), mean ± SD (range)	15.3± 4.8 (8–25)
Mean interval to the first postoperative MRI scan (months), mean ± SD (range)	4.3± 1.5 (1–10)
Mean interval between first and second postoperative MRI scan (months), mean ± SD (range)	3.8± 0.9 (2–7)
Maximum tumor diameter (mm), mean ± SD (range)	57.5 ± 17.8 (28–90)
Primary surgery	
Total resection	60 (82.2)
Subtotal resection	13 (17.8)
Tumor grade	
Grade III	31 (42.5)
Grade IV	42 (57.5)
Treatment	
Cortisone intake	28 (38.4)
Radiotherapy	73 (100)
Chemotherapy	62 (84.9)
Methods of final diagnosis	
Repeat surgical resection	27 (37)
Clinical/imaging follow-up	46 (63)
Final diagnosis	
TP	35 (47.9)
No TP	38 (52.1)

Unless otherwise indicated, data are the number of patients with percentages in parentheses. MRI = magnetic resonance imaging; SD = standard deviation; TP = tumor progression.

**Table 3:** Frequency distributions of BT-RADS categories of gliomas after the initial two postoperative MRI examinations stratified by readers and final diagnosis.

Category	R 1		R 2		R 3		R 4		R 5	
	TP	No TP	TP	No TP	TP	No TP	TP	No TP	TP	No TP
1a	1 (2.9)	6 (15.8)	0 (0)	8 (21.1)	2 (5.7)	9 (23.7)	1 (2.9)	8 (21.1)	0 (0)	10 (26.3)
1b	3 (8.6)	9 (23.7)	1 (2.9)	8 (21.1)	1 (2.9)	8 (21.1)	1 (2.9)	10 (26.3)	2 (5.7)	9 (23.7)
2	2 (5.7)	12 (31.6)	3 (8.6)	9 (23.7)	3 (8.6)	9 (23.7)	4 (11.4)	9 (23.7)	2 (5.7)	11 (28.9)
3a	5 (14.3)	7 (18.4)	3 (8.6)	8 (21.1)	4 (2.9)	6 (15.8)	3 (8.6)	8 (21.1)	1 (2.9)	3 (7.9)
3b	8 (22.9)	1 (2.6)	11 (31.4)	4 (10.5)	8 (22.9)	5 (13.2)	7 (20)	1 (2.6)	11 (31.4)	3 (7.9)
3c	5 (14.3)	2 (5.3)	10 (28.6)	1 (2.6)	8 (22.9)	1 (2.6)	6 (17.1)	2 (5.3)	3 (8.6)	1 (2.6)
4	11 (31.4)	1 (2.6)	7 (20)	0 (0)	9 (25.7)	0 (0)	13 (37.1)	0 (0)	16 (45.7)	1 (2.6)

Data are the number of gliomas with percentages in parentheses. BT-RADS = brain tumor reporting and data system; MRI = magnetic resonance imaging; R = reader; TP = tumor progression.

**Table 4:** ROC analysis for TP prediction at BT-RADS category cutoff values.

Category	Sensitivity	Specificity
≥BT-RADS-1a	100	0.0
>BT-RADS-1a	94.3–100	15.8–26.3
>BT-RADS-1b	88.6–97.1	39.5–50.0
>BT-RADS-2	68.6–88.6	65.8–79.0
>BT-RADS-3a *	68.6–85.7	84.2–92.1
>BT-RADS-3b	45.7–54.3	92.1–97.4
>BT-RADS-3c	17.1–45.7	94.7–97.4
>BT-RADS-4	0.0	100.0

Data are the range of percentages according to the reviewers. ROC = receiver operating characteristic; BT-RADS = Brain Tumor Reporting and Data System; TP = tumor progression; * = the optimal cutoff value that maximized the average of sensitivity and specificity.

**Table 5:** Diagnostic accuracy of BT-RADS for predicting TP stratified by readers.

Parameters	R 1	R 2	R 3	R 4	R 5
Cutoff	>BT-RADS-3a	>BT-RADS-3a	>BT-RADS-3a	>BT-RADS-3a	>BT-RADS-3a
True-positive findings (n)	24	28	25	26	30
False-negative findings (n)	11	7	10	9	5
False-positive findings (n)	4	5	6	3	5
True-negative findings (n)	34	33	32	35	33
Accuracy (%)	79.5 (58/73) [68.4–88.0]	83.6 (61/73) [73.1–91.2]	78.1 (57/73) [66.9–86.9]	83.6 (61/73) [73.1–91.2]	86.3 (63/73) [76.3–93.2]
Sensitivity (%)	68.6 (24/35) [50.7–83.2]	80.0 (28/35) [63.1–91.6]	71.4 (25/35) [53.7–85.4]	74.3 (26/35) [56.7–87.5]	85.7 (30/35) [69.7–95.2]
Specificity (%)	89.5 (34/38) [75.2–97.1]	86.8 (33/38) [71.9–95.6]	84.2 (32/38) [68.8–94.0]	92.1 (35/38) [78.6–98.3]	86.8 (33/38) [71.9–95.6]
Positive predictive value (%)	85.7 (24/28) [69.8–94.0]	84.8 (28/33) [70.8–92.8]	80.6 (25/31) [66.0–89.9]	89.7 (26/29) [74.2–96.3]	85.7 (30/35) [72.4–93.2]
Negative predictive value (%)	75.6 (34/45) [65.2–83.6]	82.5 (33/40) [70.6–90.2]	76.2 (32/42) [65.1–84.6]	79.6 (35/44) [68.7–87.3]	86.8 (33/38) [74.4–93.8]

Data in parentheses were used to calculate percentages. Data in brackets are 95% confidence intervals. BT-RADS = Brain Tumor Reporting and Data System; n = number; TP = tumor progression; R = reader.

**Table 6:** Inter-reader agreement for MR imaging features and BT-RADS categorization.

Readers	Features	R 2	R 3	R 4	R 5	Overall
R 1	Enhanced component	0.28 (0.15–0.40)	0.24 (0.07–0.42)	0.29 (0.13–0.44)	0.36 (0.23–0.50)	
	FLAIR component	0.60 (0.46–0.74)	0.43 (0.27–0.60)	0.56 (0.41–0.71)	0.68 (0.56–0.80)	
	Mass effect	0.62 (0.46–0.79)	0.70 (0.55–0.84)	0.64 (0.48–0.79)	0.82 (0.70–0.95)	
	New lesion	0.79 (0.59–0.99)	1.00 (1.00–1.00)	0.79 (0.51–1.00)	1.00 (1.00–1.00)	
	BT-RADS	0.57 (0.46–0.67)	0.51 (0.38–0.64)	0.54 (0.43–0.66)	0.70 (0.58–0.81)	
R 2	Enhanced component		0.33 (0.17–0.50)	0.26 (0.08–0.44)	0.65 (0.51–0.80)	
	FLAIR component		0.59 (0.44–0.73)	0.65 (0.51–0.80)	0.69 (0.57–0.82)	
	Mass effect		0.73 (0.58–0.88)	0.79 (0.65–0.93)	0.68 (0.52–0.84)	
	New lesion		0.79 (0.59–0.99)	0.65 (0.38–0.93)	0.79 (0.59–0.99)	
	BT-RADS		0.51 (0.41–0.62)	0.60 (0.49–0.70)	0.72 (0.63–0.80)	
R 3	Enhanced component			0.28 (0.11–0.45)	0.44 (0.29–0.59)	
	FLAIR component			0.46 (0.29–0.63)	0.57 (0.42–0.71)	
	Mass effect			0.71 (0.56–0.86)	0.73 (0.58–0.87)	
	New lesion			0.79 (0.51–1.00)	1.00 (1.00–1.00)	
	BT-RADS			0.54 (0.42–0.66)	0.72 (0.62–0.81)	
R 4	Enhanced component				0.46 (0.29–0.63)	
	FLAIR component				0.67 (0.53–0.81)	
	Mass effect				0.63 (0.47–0.80)	
	New lesion				0.79 (0.51–1.00)	
	BT-RADS				0.73 (0.64–0.83)	
Overall	Enhanced component					0.45 (0.34–0.56)
	FLAIR component					0.67 (0.58–0.76)
	Mass effect					0.69 (0.60–0.77)
	New lesion					0.89 (0.86–0.93)
	BT-RADS					0.75 (0.68–0.82)

Data are Kappa values. Data in parentheses are 95% confidence intervals. BT-RADS = Brain Tumor Reporting and Data System; R = reader; FLAIR = fluid-attenuation inversion recovery; MR = magnetic resonance. The overall inter-reader agreement was calculated using the intraclass correlation coefficient (ICC) with 95% confidence intervals. The inter-reader agreement between single readers was calculated using the weighted kappa statistics with 95% confidence intervals. The k values were interpreted as follows: 0.00–0.20 = poor agreement; 0.21–0.40 = fair agreement; 0.41–0.60 = moderate agreement; 0.61–0.80 = good agreement; and 0.81–1.00 = very good agreement.

**Table 7:** Reader acceptance of the BT-RADS classification system.

Questions	Score
Reporting of post-treatment glioma imaging should follow a structured format	24
Radiology reports following BT-RADS clarify the significant findings	17
The structured reporting template of BT-RADS maintains the consistency of the reports	24
The application of BT-RADS constrains radiologists’ and clinicians’ communication	23
The application of BT-RADS enables a more understandable and concise report	21
Much training is required before the application of BT-RADS	21
BT-RADS is an easily applicable system by both junior and senior radiologists	16
The application of BT-RAD needs highly experienced radiologists	21
Reporting using BT-RADS helps trainee education	20
Using a structured reporting template of BT-RADS saves time for radiologists	18
BT-RADS promotes confidence in the final categorization of post-glioma imaging	16
BT-RADS enables the determination of appropriate management strategy	19
Some post-glioma imaging was difficult to interpret by BT-RADS	22
BT-RADS is equivalent to other ACR reporting systems, such as BI-RADS and LI-RADS	14
The current BT-RADS system is satisfied	15
BT-RADS needs further modification	24
The application of BT-RADS should be encouraged	25

The score represents the sum of points provided by each reviewer. BT-RADS = Brain Tumor Reporting and Data System; BI-RADS = Breast Imaging Reporting and Data System; LI-RADS = Liver Imaging Reporting and Data System; ACR = American College of Radiology. To assess reviewer acceptance of BT-RADS, all reviewers completed a short survey after achieving their review. Answers were provided on an ordinal scale of 5 points. The scores were calculated as the sum of each reader’s points.

## Data Availability

The clinical and imaging datasets used and/or analyzed during the current study are available from the corresponding author upon reasonable request.
